# Bridging gaps: a systematic literature review of brokerage in educational change

**DOI:** 10.1007/s10833-023-09493-7

**Published:** 2023-10-07

**Authors:** Beat Rechsteiner, Eva Kyndt, Miriam Compagnoni, Andrea Wullschleger, Katharina Maag Merki

**Affiliations:** 1 Institute of Education, University of Zurich, Freiestrasse 36, 8032 Zurich, Switzerland; 2 Centre for the New Workforce, Swinburne University of Technology, Melbourne, Australia; 3 University of Applied Sciences and Arts Northwestern Switzerland, Windisch, Switzerland

**Keywords:** Brokerage, Broker, Brokering, Educational change, Knowledge mobilization, Capacity building

## Abstract

Bridging gaps between educational stakeholders at the classroom, school, and system levels is essential to achieve sustainable change in primary and secondary education. However, transferring knowledge or building capacity within this network of loosely coupled stakeholders is demanding. The *brokerage* concept holds promise for studying these complex patterns of interaction, as it refers to how specific actors (*brokers*) link loosely coupled or disconnected individuals (*brokering*). However, different research traditions, in terms of theoretical frameworks and methodological approaches, and various stakeholders examined in their role as bridge builders make understanding the role of brokers, brokering, and brokerage in changing educational practice challenging. Therefore, the purpose of this study is to provide an overview of the current literature on these concepts in educational change research. In a systematic literature review based on 42 studies, we analyzed each study’s theoretical assumptions, methodological approach, scope in terms of stakeholders involved, and empirical findings. First, the literature review revealed that research on educational change refers to four different theoretical frameworks when focusing on brokers, brokering, or brokerage. Second, our results indicate that predominantly qualitative approaches have been applied. Third, using content network graphs, we identified teachers and principals as among the most frequently analyzed brokers. Fourth, four relevant aspects of the empirical findings are presented: brokers’ personal characteristics, conditions that enable brokering, successful brokering strategies, and outcomes of brokerage. Finally, we outline a future research agenda based on the empirical evidence base and shortcomings.

## Introduction

Almost 50 years ago, Weick (1976) coined the notion that educational organizations are loosely coupled systems. Recent research has shown that this still holds true (Meredith et al., 2017; Shen et al., 2017). Thus, when trying to improve educational practice, various professional stakeholders (i.e., teachers, principals, and policymakers) pursue interests that are only sometimes aligned. These loose coupling mechanisms may lead to gaps emerging—not only between different stakeholders on the class, school, and system level but also between other actors more at the periphery of the school system, such as researchers or professional intermediaries. Bridging these gaps is not trivial, making changing educational practices in schools demanding.

However, change is vital for schools, as conditions and requirements for schools are constantly changing. Nevertheless, sustainable improvements at the core of educational practice are more the exception than the rule (Hargreaves et al., 2014; Mitchell & Sackney, 2011; Tyack & Tobin, 1994). Thus, scaling up these exceptions of high-quality school improvement to a broader educational landscape, be it on a class, school, or system level, seems often equivalent to squaring the circle (Elmore, 1996; Fullan, 2016). Bryson et al. (2015) indicated that complex public issues could only be resolved when stakeholders with different backgrounds, knowledge, and agendas know how to and are willing to collaborate. Hence, to provide learning environments that adapt to the needs of all students, it is essential to know how emerging gaps between various stakeholders can be bridged so that collaboration and professional development is enhanced (Kyndt et al., 2016).

According to social network theorists (i.e., Burt, 1992; Gould & Fernandez, 1989), the concept of *brokerage* offers a theoretical and analytical lens to understand better how gaps can be successfully bridged to improve practice on an individual and collective level. From a social network perspective, brokerage can be defined as the dynamic interplay of actors when controlling and organizing the flow and content of information (Borgatti & Foster, 2003). Outside the school context, the concept has been studied extensively and investigated systematically within various research areas, such as in public health (Lomas, 2007; Long et al., 2013), management research (Kwon et al., 2020), and sociology (Stovel & Shaw, 2012).

However, brokerage may also play a crucial role in the loosely coupled educational context, specifically in research on educational change. When we searched for studies explicitly applying brokerage as a theoretical lens in research on educational change, we soon realized that the field is challenged by conceptual confusion. In the following, we outline three aspects of how this confusion manifests and what consequences this may have on research in the domain of educational change:

First, no single brokerage concept exists but rather multiple frameworks (also referring to brokers and brokering) that originated in different research traditions are common. Thus, there might be different understandings about brokerage.

Second, the studies employed various methodological approaches when identifying and analyzing brokerage. As a result, there are not only multiple conceptualizations, but also different operationalizations of how brokerage is assessed.

Third, deducing key findings and insights for educational change from the available empirical research seems challenging. A lack of conceptual clarity makes it immensely difficult to establish common ground on what we already know about brokerage regarding educational change. Thus, although there are attempts to transfer the concept to the school context, it remains unclear whether they have developed more in isolation rather than linked to each other. This lack of common ground (conceptually and methodologically) may have hampered researchers from using the concept more prominently in research about educational change.

Hence, this study aims to systematically review the current literature on the concept of brokerage in research on educational change by clarifying theoretical assumptions and methodological approaches, systematically mapping the stakeholders analyzed as brokers, and listing the essential findings. Finally, based on the shortcomings of those insights, a future research agenda is outlined.

### Conceptualizing brokers, brokering, and brokerage

From an etymological standpoint, the expression broker originates in the middle English word *brokour*, akin to the Spanish *alboroque*, which stands for a ceremonial gift at the conclusion of a deal (American Heritage Dictionary, 2020). Historically, a broker makes profits by negotiating goods or ideas and coordinating contacts between communities otherwise disconnected. Three terms are closely related to this conception and are often used either synonymously or as overlapping terminologies: broker, brokering, and brokerage. In this paper, we refer to *brokers* as individuals or groups acting as intermediaries, *brokering* to activities these actors apply when working the interface (Meyer, 2010), and *brokerage* as the dynamic and complex set of actors (brokers) and activities (brokering) involved in negotiation processes between distinct social worlds (Stovel & Shaw, 2012). In the following, we refer to the term *brokerage* when both brokers and brokering or the relation between the two are addressed.

There is a long tradition of conceptualizing and analyzing brokerage in social networks based on social capital theories (i.e., Burt, 1992; Marsden, 1982). Social capital theories with an explicit focus on *bridging capital* (Putnam, 2000) provide a theoretical lens to explore how social relationships impact the flow of information (Crossley et al., 2015). Based on the assumption that social capital is not equally distributed among individuals, a brokerage position within social networks impacts an individual’s leverage to control the flow of information and access otherwise disconnected individuals in a broader, more diverse network (Marsden, 1982). In addition, a critical assumption is that social capital is not only determined by formal roles and organizational structures but also (and foremost) by informal ties between actors (Burt, 2005). Following these assumptions, social network analysis techniques are most frequently used to locate stakeholders in brokerage positions, examine to what degree formal and informal network structures correspond, and study different patterns of behavior due to inequalities in social capital (Crossley et al., 2015).

However, systematic reviews on the brokerage concept outside the school context have indicated that, depending on the field of application, research traditions beyond social capital theories and social network analysis have conceptualized and examined brokerage (i.e., Kwon et al., 2020; Long et al., 2013). For example, in research on knowledge mobilization, researchers have applied the brokerage concept to better understand why individuals and organizations differ in how they can effectively combine know-hows, know-whys, and know-whos (Meyer, 2010; Ward et al., 2009). These different research traditions are also associated with different methodological approaches. For example, unlike social capital theories, research on knowledge mobilization relies primarily on qualitative methods (i.e., Kwon et al., 2020; Lomas, 2007; Ward et al., 2009).

Consequently, the question arises as to which theoretical framework is applied when brokerage is examined in research on educational change. A domain-specific overview of all applied frameworks and the associated various methodological approaches can contribute to clarifying the conceptual confusion and allow us to understand better the complex dynamics and constraints in the interaction patterns of different stakeholders involved in brokering—for example, in terms of the ebb and flow of information in a school’s social network (Borgatti & Foster, 2003). However, in the next section, we want to further elaborate on the importance of studying brokers, brokering, and brokerage to understand better how emerging gaps may be bridged to change educational practice.

### Bridging gaps for educational change

There are several reasons why brokers might be in critical positions when it comes to solving problems and implementing innovative ideas not only in private companies (i.e., Burt, 2005; Obstfeld, 2005) but also in public organizations (i.e., Lomas, 2007; Ward et al., 2009) such as schools.

By mobilizing knowledge across emerging gaps, brokers have immediate access to non-redundant information and innovative ideas. Thus, brokers may disrupt the tendency of reproduction as they introduce new perspectives on daily practices across gaps emerging between different communities and organizations (Burt, 2005).

Accordingly, research has found that brokers filter, distort, or hoard resources, which may provide benefit in the form of control or power to the broker but may simultaneously inhibit overall individual and organizational performance (i.e., Burt, 1992, 2005; McGrath & Krackhardt, 2003). Thus, on the dark side, brokers can also misuse their privileged position for their interests (i.e., Krackhardt, 1999). Moreover, being in a sandwich position between different groups can, in the long run, be very burdensome and associated with feelings of isolation since not belonging to one group or the other (i.e., Carboni & Gilman, 2012; Mollenhorst et al., 2015). Hence, too much weight on their shoulders may also inhibit sustainable improvements.

Furthermore, schools are complex organizations making communication and knowledge transfer among staff members an intricate and challenging endeavor (Tortoriello et al., 2012). Thus, the gaps that need to be bridged can also occur within organizations (for instance, horizontally between subgroups or vertically between leaders and subordinates), where over time and due to different reasons, communities may distance themselves from each other, leading to a potentially widening gap. The brokerage concept may help to illuminate the complex interaction patterns within schools when changing educational practice.

Eventually, regarding changing educational practice, researchers have pointed out that fundamental conceptions of learning and teaching remain relatively static (Bryk et al., 2011; Tyack & Tobin, 1994). Nevertheless, there are good examples—in terms of high-quality teaching, effective school improvement programs, or valuable research evidence—of how and in what direction educational practice should be changed (i.e., Kyriakides et al., 2019). Unfortunately, these examples too often remain isolated islands unattached to the broader educational landscape (Elmore, 1996; Hubers, 2020). Therefore, it is often not a problem of the supply of good examples but of deficiencies in how this knowledge is transferred or adapted to the school’s context (Elmore, 1996; Malouf & Taymans, 2016). That is where the brokers may come in.

In the following, we elaborate on how various stakeholders involved in changing educational practice may act as brokers building bridges, for better or worse, in the complex web actors on the class, school, and system level.

#### Actors on the class and school level

First, having the most direct impact on student learning, teachers are crucial when it comes to changing educational practice. For instance, teachers may experiment with new materials or innovative ideas and subsequently implement what has proven to work in their local context (Mitchell & Sackney, 2011). Teachers change their practice the most effectively, not in isolation but collaboratively (Horn et al., 2020; Sinnema et al., 2021)—for instance, in professional learning communities (Louis et al., 1996; Mitchell & Sackney, 2011). Thus, teachers constantly build bridges with other actors within and outside their school—i.e., colleagues, principals, social workers, or special needs teachers. These relations may be productive or conflictual.

Second, school principals, as actors on the school level, have a more indirect impact on student learning (Creemers & Kyriakides, 2007), as they may foster or hinder an environment in which teachers have the resources and support to develop educational practice further (Leithwood et al., 2020). Thus, principals may function as intermediaries—within and outside their school. In this way, they may exchange ideas on improving organizational structures and teachers’ pedagogical repertoires (Kolleck, 2016). Moreover, Louis and Dentler (1988) have pointed out that the diffusion of knowledge in schools is most significant when an indirect strategy is applied. Hence, applying a brokerage lens might help to analyze the who, what, and how of these indirect paths that knowledge ‘travels’ when actors on the class and school level are about to change their educational practice.

#### Actors on the system level

Apart from this inner circle of actors, on a system level there are policymakers, district leaders, superintendents, curriculum coordinators, or administrators working within and organizing the broader political landscape of an educational system. In the following, we refer to this group as central office staff. These actors may set directions, support actors on the class and school levels, and coordinate collaborative action between schools or other school systems (DuFour, 2003; Honig, 2006). In this way, they shape the focus and leeway of principals and teachers in their professional development. Furthermore, according to Honig and Hatch (2004), collaboration between schools and school districts depends on the stakeholders’ skills in crafting coherence about what conditions are necessary, what processes are effective, and what outcomes are desirable when changing educational practice. Thus, analyzing brokerage between the school and system level might lead to a better understanding of what factors foster or hinder stakeholders from aligning perspectives regarding changing educational practice.

#### External experts

Last, there are actors involved in educational change who are more peripheral to the educational systems, such as researchers, professional intermediaries, or teacher educators. Seeking to change educational practice through more evidence-based reform (Slavin, 2020), external experts, such as researchers or science communicators, have gained influence on improving educational practice (Ball & Junemann, 2012; Lubienski et al., 2014; Malin et al., 2018). For instance, Honig and Ikemoto (2008) indicated that external experts may act as adaptive assistants in system-wide improvement efforts when the needs of district and school-level stakeholders must be met. However, particularly in the case of professional intermediaries, these new connections may lead to a substantial shift in the actor constellation. Thus, Lubienski et al. (2014) pointed out that professional intermediaries operate in new, poorly analyzed ways, making research–practice-partnerships and policy networks more fluid. The brokerage concept may help to examine further whether these new emerging power structures within educational systems solve or even exacerbate pending issues regarding educational change.

By systematically reviewing the literature on brokers, brokering, and brokerage in educational change, we can examine the extent to which the stakeholders outlined above act as bridge builders when educational practice develops. Moreover, other stakeholders acting as brokers (sometimes hidden or unexpected) may be identified that have not yet been in our focus. Eventually, providing an overview of the empirical evidence base for each stakeholder makes it possible to not only better understand social interaction patterns and dynamics within and between schools and other stakeholders involved in changing educational practices but also to outline a future research agenda outlining where to go next.

### Present study

Systematizing the knowledge base and conceptualization of brokerage, brokers, and brokering strategies in educational change literature is essential to establish a common ground and bring the brokerage concept more prominently into the discussion. In addition, it might set the stage for further developing the research on building bridges among loosely connected actors involved in educational change.

This study addresses the following questions using the selected studies on brokerage, brokering, and brokers in educational change:

- RQ1: What theoretical frameworks did the studies adopt?
- RQ2: What methodological approaches were used?
- RQ3: What stakeholders involved in brokering activity were analyzed?
- RQ4: What evidence on improving educational practice did they find?

## Methods

A systematic literature review (Xiao & Watson, 2019) was conducted in four consecutive phases: First, scientific literature was searched in several databases. Second, relevant articles were selected based on criteria for inclusion starting from these search results. Third, the articles were critically appraised in order to exclude studies of low quality. Finally, the studies were analyzed using a mixed-method synthesis approach (Heyvaert et al., 2013, p. 659), combining qualitative content and network analysis.

### Literature search

**Fig. 1:**
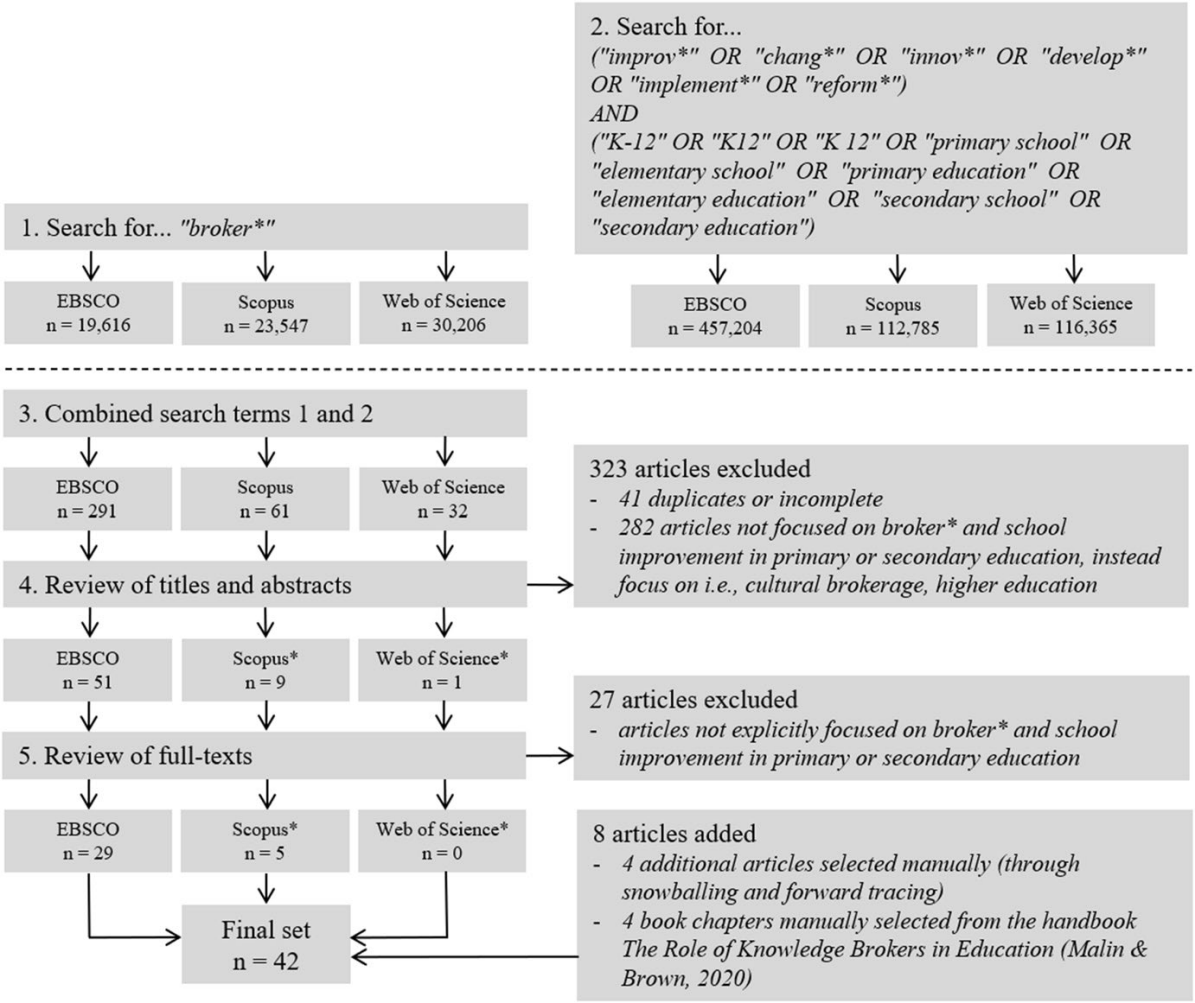
Flow chart of search strategy for literature review. Only unique articles not in EBSCO results. Final literature search was conducted in March 2023

In the first phase, the relevant literature on brokerage in research about educational change was searched. As brokerage is an interdisciplinary concept, different relevant databases within EBSCO were consulted: ERIC for research in education, EconLit for research in economics, PsycARTICLES and PsycINFO for research in (organizational) psychology, and SocINDEX for research in sociology. In addition, we looked for relevant literature in other interdisciplinary databases such as Scopus (Elsevier) and Web of Science (Social Science Citation Index)—also covering scientific publications from the health sector where the brokerage concept has been widely discussed already. The search terms for brokerage and educational change were introduced in the first and second steps (see Fig. 1). We applied the search term broker* to cover all three search terms: broker, brokering, and brokerage. As educational change is an umbrella concept with different facets, such as professional development or school improvement through the implementation of innovations and reforms, we used a variety of keywords related to change: improvement, innovation, development, implementation, and reform.

This search resulted in about 20,000–30,000 articles in each database for broker* (see step 1 in Fig. 1) and a range of 112,785 (Scopus) to 457,204 (EBSCO) articles for educational change in primary and secondary school (see step 2 in Fig. 1). In the third step, the different search terms were combined (see step 3 in Fig. 1). Combining the search terms resulted in 384 articles across all databases. Our selection was limited to English-language publications only. Apart from that, we applied no further criteria for automatic elimination.

### Criteria-based literature selection

The second phase was conducted in multiple steps: First, we selected articles by screening the title and abstract—highlighting relevant keywords related to our inclusion criteria, and removing duplicates (see step 4 in Fig. 1). After this step, 61 articles remained. Second, we read the full texts of these remaining articles to determine their primary research focuses (see step 5 in Fig. 1). Third, four raters were involved in testing the reliability of the selection criteria. For this, 10 out of the remaining 61 articles from the pre-selection were randomly picked, and four questions based on the selection criteria listed above were applied:

- Is there a clear focus on changing educational practice?
- Are brokers, brokerage, or brokering mentioned as a central issue in the publication?
- Does the brokering activity occur between professional members or associates of the educational system (not with pupils or parents)?
- Was the research conducted in the context of primary or secondary education?

Each rater answered the four questions with ‘true’ or ‘false.’ An article was included if all of the four statements were found to be true. The interrater reliability of the different selection criteria was measured using Krippendorff’s alpha (Krippendorff, 2011). An alpha value of 0.71 among the raters revealed that the set of criteria was reliable to apply these selection criteria to all of the other remaining 51 articles that initially resulted in the literature search. Eventually, based on reading the full texts, 34 articles were identified where the scope and emphasis of the studies matched the selection criteria. Third, backward and forward iterations in the analysis of the citations of the retrieved articles resulted in the inclusion of 8 additional studies, leading to a total of 42 selected articles.

### Critical appraisal

In the third phase, the quality of the 42 articles included in the final selection was assessed using the critical appraisal tool CASP (Critical Appraisal Skills Programme, 2013) as adopted by Kyndt et al. (2016). The main criteria were: (1) “a well-focused research question,” (2) “an appropriate research design” in terms of matching methodological approach to the research questions, (3) “a well-described and appropriate sampling strategy, data collection, and analysis method,” and (4) “a clear description of the research findings” (see Kyndt et al., 2016, p. 1119). No low-quality studies were identified in our selection, and we included all 42 studies in the analysis.

### Analysis of literature

In the last phase, to analyze data from these 42 articles, we applied a mixed method strategy in three successive steps to answer our research questions: In the first step, study characteristics were inventoried (e.g., language, region). In the second step, using a content analysis approach (Miles & Huberman, 1994), data were extracted on the following subtopics: the theoretical assumptions on which brokerage, brokers, or brokering were based (RQ1), the methodological approach applied (RQ2), the stakeholders involved (RQ3), and the empirical findings on brokerage (RQ4). Finally, regarding the studies’ methodological approaches, we analyzed both study design (such as data collection and analysis) and how brokers, brokering, and brokerage was identified or measured.

In a third step, we conducted content network analyses to process the various stakeholders involved in brokering activities (RQ3) and to map the current empirical evidence base (RQ4). This new approach to systematic reviews allowed us to present complex information in lucid graphs, which facilitates the interpretation of interrelations between stakeholders, approaches, and perspectives. *Content networks* have been defined as networks in which nodes represent content and ties indicate the co-occurrence of that content (Rice & Danowski, 1993). For our analyses, we used both directed and undirected ties: Directed ties in a content network graph indicate in which direction the connection between two nodes runs. Undirected ties indicate associations between different aspects. In both cases, the weight of the ties represents the number of connections between two specific nodes. For content networks based on directed ties, the size of the nodes depends on the out-degree centrality, a network measure (Freeman, 1977) based on out-going ties. The size of nodes in the content networks with undirected ties was computed based on degree-centrality. The higher the degree-centrality, the larger a node and the more it is connected to other nodes in the network. All the network graphs were made using the *igraph* package Version 1.3.5 (Csardi & Nepusz, 2006) in R (RStudio Team, 2020).

## Results

In the following, we first present the general characteristics of the studies selected and then address the research questions on theoretical frameworks (RQ1), methodological approaches (RQ2), and which stakeholders have been analyzed (RQ3). In the final section, we present the current research base of empirical evidence on brokerage in educational change (RQ4). Table 1 provides detailed insights into the findings for each selected study separately.

**Table 1:** Theoretical frameworks, research topics, methodology, and empirical evidence of the selected studies

Author(s)	Year	Theoretical framework	Methodology					Stakeholders	Empirical evidence
			Study design	Data collection	Sample size	Ack. of time	Measurement		
Rechsteiner et al	2022a	Soc. Learn	Quant	2, 3	Large	Cross-sect	–	1, 2	1, 2, 4
Rechsteiner et al	2022b	Soc. Capital	Quant	2, 3	Large	Cross-sect	Betw	1, 2	1, 2
Van den Boom-Muil. et al	2022	KMb	Mixed	1, 3	Large	Time-lagged	Betw	1, 2	1, 2, 3, 4
Fahy & Kenny	2022	Soc. Learn	Qual	1	Small	Cross-sect	–	1, 7	2, 3, 4
Jusinski	2021	Soc. Learn	Qual	1, 2, 4	Small	Time-lagged	–	1	1, 2, 3, 4
Hashim	2020	Soc. Capital	Mixed	1, 3	Medium	Cross-sect	Betw., Formal	3	3, 4
Chang	2020	KMb	Qual	1, 4, 5	Small	Time-lagged	–	3, 4	3, 4
Farley-Ripple & Grajeda	2020	KMb	Mixed	1, 2, 3	Medium	Cross-sect	–	1, 2	1, 2, 3, 4
Cooper et al	2020	KMb	concept	-	-	-	–	4	-
Brown	2020	KMb	Quant	2, 3	Large	Cross-sect	–	1	1, 2, 3
Davidson & Penuel	2020	Soc. Learn	Qual	1	Medium	Time-lagged	–	3	1, 3
Hopkins et al	2019	KMb	Quant	2, 3	Medium	Cross-sect	Formal	3, 4, 5	1, 3
Neal et al	2019	KMb	Quant	3	Large	Cross-sect	Formal	1, 3, 5	2, 3, 4
Cooper	2019	Soc. Learn	Qual	4	Medium	Cross-sect	–	1, 5	1, 3, 4
Van Gasse et al	2019	KMb	Qual	1	Small	Cross-sect	–	1, 2, 4	1, 2, 3, 4
Morel & Coburn	2019	KMb	Mixed	1, 3	Medium	Cross-sect	Betw., ERGM	3, 4	1, 2, 4
Hopkins et al	2018	KMb	Quant	2, 3	Medium	Cross-sect	Betw	3, 5	1, 3, 4
Zuckerman et al	2018	Soc. Capital	Qual	4	Small	Time-lagged	–	2	3, 4
Hubers et al	2018	KMb	Mixed	3, 4	Medium	Longitudinal	Formal	1, 2	3, 4
LeChasseur et al.	2017	KMb	Qual	1	Medium	Time-lagged	–	2	3
Wilkie	2019	KMb	Qual	1, 2	Medium	Time-lagged	–	1, 5	1, 2, 3
Malin & Paralkar	2017	KMb	Qual	1, 2, 5	Medium	Cross-sect	–	4	1, 3, 4
Nordholm	2016	Soc. Learn	Qual	1, 6	Small	Time-lagged	–	1, 2	1, 2, 3, 4
Willegems et al	2016	KMb	Qual	4, 6	Small	Time-lagged	–	6	1, 2, 4
Durand et al	2015	Dist. Leader	Mixed	1	Small	Cross-sect	–	3	3
Ng-A-Fook et al	2015	KMb	Mixed	1, 2	Medium	Time-lagged	–	1, 6	1, 2
Sharples & Sheard	2015	KMb	Qual	1, 2	Large	Cross-sect	–	1, 2, 6	1, 2
Daly et al	2014a	Soc. Capital	Quant	2, 3	Large	Cross-sect	Betw	2, 3	1, 2, 3, 4
Daly et al	2014b	Soc. Capital	Quant	2, 3	Large	Cross-sect	Betw	2, 3	1, 2, 3, 4
Jabbar et al	2014	KMb	Qual	1, 4	Medium	Cross-sect	–	3, 4	1, 3, 4
Hopkins et al	2013	Soc. Capital	Mixed	1, 2, 3	Large	Longitudinal	Betw	1, 2, 3	1, 2, 3, 4
Slavit & Roth McDuffie	2013	Dist. Leader	Qual	4, 5	Medium	Time-lagged	–	1	1, 2, 3, 4
Cooper	2012	KMb	Mixed	5	Medium	Cross-sect	–	1, 2	1, 3, 4
Spillane & Kim	2012	Soc. Capital	Mixed	2, 3	Large	Longitudinal	Betw	1, 2	1, 2, 4
Wong	2012	Soc. Learn	Qual	1, 4	Small	Time-lagged	–	1, 6	3, 4
Kisiel	2010	Soc. Learn	Qual	1	Medium	Time-lagged	–	1, 7	1, 4
Park & Datnow	2009	Dist. Leader	Qual	1, 4, 5	Medium	Cross-sect	–	2, 3	2, 3
Kubiak	2009	Soc. Learn	Qual	1, 4, 5	Small	Time-lagged	–	2, 3, 4	1, 2, 3
Supovitz	2008	KMb	Qual	1	Medium	Time-lagged	–	3, 4	3, 4
Swinnerton	2007	Dist. Leader	Qual	1, 4	Small	Time-lagged	–	3	2, 3, 4
Carmichael et al	2006	Soc. Capital	Qual	3	Medium	Cross-sect	–	1, 2, 3	1, 3, 4
Corbin et al	2003	Soc. Learn	Qual	1, 4	Small	Time-lagged	–	1, 2, 3	2, 3

*Theoretical frameworks*: KMb = knowledge mobilization frameworks, Dist. Leader = distributed leadership, Soc. Capital = social capital theories, Soc. Learn = social learning theory; *Stakeholders*: 1 = teachers, 2 = principals, 3 = central office staff, 4 = professional intermediaries, 5 = researchers, 6 = teacher educators, 7 = Others; *Study design*: Qual = qualitative research, Quant = quantitative research, Mixed = mixed methods, Concept = conceptual paper; *Data collection*: 1 = interview; 2 = survey, 3 = social network data, 4 = observation, 5 = documents, 6 = logfiles; *Sample size*: Small = N < 20, Medium = 21 < N < 120, Large = N > 120; *Ack. of time* (acknowledgment of time): Cross-sect. (cross-sectional), Time-lagged, or Longitudinal data; *Measurement*: Betw. = betweenness-centrality, Formal = brokerage typology based on the formal approach by Gould and Fernandez (1989), ERGM = exponential random graph models; *Empirical evidence:* 1 = personal characteristics; 2 = conditions; 3 = strategy use; 4 = outcomes

### General characteristics of the selected studies

A closer look at the studies’ characteristics revealed two initial findings (see Table 2): First, most studies were conducted in North America (*n* = 26), followed by Europe with 13 studies. Only a few studies (*n* = 3) were conducted elsewhere. Second, the earliest publication only went back to 2003, indicating that the discussion on brokerage in educational change is relatively new. Earlier publications addressed the brokerage concept in the domain of educational science, but they did not examine educational change; they dealt with teachers as brokers between scholarly knowledge and their pupils (i.e., Sabatino, 1982; White, 1987) or with *cultural brokerage* to link school and families with different ethnical backgrounds (i.e., Gentemann & Whitehead, 1983; Stairs, 1995).

**Table 2:** Descriptive information on the selected studies

Criterion	Category	*N*
Region	North America (i.e., United States, Canada)	26
	Europe (i.e., United Kingdom, Belgium, the Netherlands)	13
	Asia (i.e., China)	2
	Australia	1
	Others	0
Year of publication	After 2015	24
	2010–2015	12
	Before 2010 [oldest publication published in 2003]	6
Type of document	Peer-reviewed article in a scientific journal	35
	Book chapter	6
	Dissertation	1
	Other	0

### Theoretical frameworks (RQ1)

Although there is a generality of what brokers are and what brokerage is about, the meaning of brokerage has always depended on theoretical frameworks chosen by researchers and thus differs among studies (Corbin et al., 2003). Table 3 lists these frameworks next to each other to indicate similarities and differences in theory base, conceptualization, significant components such as conditions, processes, and outcomes, and related theoretical frameworks regarding educational change literature. Our analysis of the studies identified four theoretical anchors: (1) brokerage and brokers in social capital theories, such as the structural holes theory (Burt, 2005), (2) brokering and brokers in knowledge mobilization frameworks originating in bridging the research-practice gap (i.e., Lomas, 2007; Ward et al., 2009), (3) brokering in Wenger’s (1999) social theory of learning on “communities of practice,” and (4) brokers in distributed leadership theories in education (i.e., Spillane, 2005).

**Table 3:** Overview of brokerage theories and their characteristics

Name of theoretical framework	Social capital theories	Knowledge mobilization frameworks	Social learning theories—communities of practice	Distributed leadership theory
Relevant publications	Burt (1992), Gould and Fernandez (1989)	Lomas (2007), Meyer (2010), Ward et al. (2009)	Wenger (1999)	Spillane (2005)
Typology	Brokerage, Broker	Brokering, Broker	Brokering	Brokers
Theory base	Social network theories to explore formal and informal network structures and their impact on the flow of information	Frameworks to analyze research-practice partnerships	Socio-constructivist theory to analyze collective and organizational learning processes	School leadership theory in which responsibilities to organize and further develop school is shared among colleagues
Conceptualization broker, brokerage, or brokering	*Broker* as an actor connecting otherwise disconnected actors in a social network *Brokerage* as a form of social capital (power, control, etc.)	Knowledge *brokers* as individuals applying knowledge *brokering* strategies to transfer knowledge from research to practice	Negotiation of boundary objects through *brokering* strategies between different communities of practice	Organizational structures such as middle leaders or teacher leaders acting as *brokers*
Major components Conditions Processes Outcomes	Structural holes in a social network Controlling the flow of information by filtering, distorting, and hoarding resources Having access to non-redundant information, agents of change	Knowledge is treated as an action rather than a commodity Knowledge management, linking agency, and capacity building Effective knowledge transfer and implementation	Brokers as conversant in the discourse of more than one community Building bridges, translating, coordinating, and aligning perspectives Create overlap, shared space to negotiate boundary objects	Formal or informal leadership roles Connecting different sub-teams in a school as a multiplicator or facilitator Foster knowledge sharing and capacity building processes among colleagues
Related theoretical frameworks in literature about educational change	Social capital in teachers’ professional interactions (Penuel et al., 2009)	Research-practice partnerships (Coburn, & Penuel, 2016; Farley-Ripple, 2019)	Professional learning communities (Louis et al., 1996)	Middle leadership (Harris et al., 2019); teacher leadership (Robinson 2008)
Example in selected studies	Daly et al. (2014a, b)	Malin and Paralkar (2017)	Kisiel (2010)	Park et al. (2009)

#### (A) Social capital frameworks

The first theoretical framework we looked for was theories related to social capital. Indeed, we found studies related to assumptions about social capital (*n* = 8). One prominent social capital theory on brokerage is the structural holes theory (Burt, 1992), which has been applied in several studies. Burt states that a structural hole occurs when two actors are not directly connected. A broker connects otherwise disconnected actors and bridges such a structural hole—i.e., district leaders in the study by Daily, Finnigan, Jordan, et al. (2014), who are supposed to act as brokers to support weak schools. In that perspective, social capital is a function of brokerage across structural holes. In addition, there was a second highly influential social capital theory applied in the selected studies: the theory of the strength of weak ties (Granovetter, 1973). Although not explicitly about brokerage, the theory’s basic assumption is that having weak connections to individuals outside the closer social network gives actors access to non-redundant information and consequently an information advantage over others in their network who are more embedded in a network of strong ties.

#### (B) Knowledge mobilization frameworks

In our case, most of the selected studies did not frame their theoretical assumptions based on a social capital theory but instead within a knowledge mobilization theory (*n* = 20). This second theoretical thread is a conglomerate of related frameworks rather than a single grand theory. Knowledge mobilization frameworks have two things in common: First, knowledge is treated as an action rather than a commodity (Sfard, 1998), and second, these frameworks have their origin in the health sector, where the challenge of moving knowledge from research into practice has been studied for quite some time (for an overview, see Lomas, 2007). One of the most influential conceptual pieces, often cited in the selected articles, is Ward et al.’s (2009) article, “Knowledge Brokering: The Missing Link in Evidence to Action Chain?” According to Ward et al., the purpose of knowledge brokerage is “to make research and practice more accessible to each other” (Ward et al., 2009, p. 2). Thus, successful brokering involves finding anchors in different communities to transfer knowledge by applying activities such as linking agency, information management, and capacity building (Ward et al., 2009). Following this argumentation, Malin and Paralkar (2017) analyzed how influencers broker educational research, news, and ideas to practitioners.

#### (C) Social learning theory

The social theory of learning *communities of practice* (Wenger, 1999) was the third framework applied in the selected studies (*n* = 10). This socio-constructivist framework focuses on negotiating boundary objects (such as innovative ideas or helpful insights to solve a problem) through brokering strategies between different communities of practice (such as organizations or working groups). At the core of this concept is the idea of a boundary, conceptualized as sociocultural differences between communities of practice. When confronted with these differences, actors see their work, or better their practice, in a new light. Boundary objects play a crucial role in overcoming differences and learning from each other. To this end, brokers may apply strategies such as translating, coordinating, and aligning various perspectives and ideas from one community to another. The ultimate goal of these activities is to build a fabric of social agencies that facilitates lifelong learning. According to Wenger (1999), brokers play a crucial role in facilitating connections between and working at the interface of communities of practice. Thus, this framework defines brokers as conversant in the discourse of more than one community (Cooper et al., 2020). Brokers can take advantage of their multi-membership by controlling the transfer of elements of one practice into another (Wenger, 1999). However, being pulled in to become full members and simultaneously being rejected as intruders, brokers must carefully manage the coexistence of membership and non-membership (Wenger, 1999). These tensions can be best summarized as a balancing act, as brokers need to keep enough distance to contribute valuable knowledge by adding a different perspective but also be aware that by distancing from the core of a community, their legitimacy to be listened to attentively declines (Wenger, 1999). An example in our literature selection referring to this theory was a study by Kisiel (2010) on collaboration between a school and a local aquarium; the study analyzed how the communities managed (and struggled) to create a shared space in which both practices could develop their practices successfully.

#### (D) Distributed leadership

A minority of the selected studies addressed issues of distributed leadership to foster an environment of constant change (*n* = 4). This last theoretical framework (Spillane, 2005) applied in the selected studies is a concept that is not directly linked to brokerage. However, the authors of these studies pick up this terminology to offer valuable insights on how various connections of shared leadership practice to brokerage assumptions can be drawn. For instance, it was assumed that middle or teacher leaders might act as brokers, facilitators, or multiplicators, connecting different sub-teams in a school, and are crucial to knowledge-sharing and capacity-building processes (Slavit & Roth McDuffie, 2013; Swinnerton, 2007).

Referring to this approach, Park and Datnow (2009) illustrated that leadership issues cannot be analyzed only at the individual level. Instead, collective aspects must also be considered, e.g., the extent to which team members are involved in decision-making, and how formal and informal organizational structures complement or contradict each other.

### Methodological approaches (RQ2)

The selected studies’ methodological approaches (Table 4) are outlined below in two consecutive steps: (1) based on the study design categorized as a quantitative, qualitative, or in a mixed method approach, and (2) based on the way that brokers and brokering activities were identified or measured.

**Table 4:** Descriptive Information on the selected studies’ theoretical frameworks, research focuses, and applied methodological approaches

Criterion	Category		*N*
Theoretical background	Social capital theories		8
	Knowledge mobilization frameworks		20
	Social learning theories		10
	Distributed leadership theory		4
Study design	Qualitative		23
	Quantitative		9
	Mixed method		9
	Conceptual		1
Acknowledgment of time	Cross-sectional		22
	Time lagged		17
	Longitudinal		3
Sample size	Small sample (N < 20)		13
	Medium sample (N = 21–120)		16
	Large sample (N > 120)		11
Level of brokerage	Within-level (n = 27)	System level	10
		School level	8
		Class level	9
	Cross-level (n = 15)	Between system & school level	9
		Between school & class level	5
		Across all levels	1
Stakeholders involved in brokering activity^a^	Teachers		32
	Principals		31
	Central office staff		26
	*External intermediaries*		
	Professional intermediaries		20
	Researchers		5
	Teacher educators		10
	Others		2

^a^In a single study, multiple stakeholders could have been addressed

#### Study design

Most of the selected studies adopted a qualitative study design (*n* = 23) (see Table 4). These studies often applied a case study methodology with small to medium-sized samples, and they relied on interview data and observations. Data analyses were conducted using open coding approaches (Miles & Huberman, 1994) or grounded theory (Strauss & Corbin, 1997). A growing number of quantitative studies (*n* = 9) used survey and social network data to examine brokerage. Both formal and informal brokerage were analyzed by applying linear regressions, structural equation modeling techniques, and social network analyses in medium to large samples.

Moreover, mixed-method studies combined and triangulated qualitative and quantitative data (*n* = 9). Some of these studies included social network analyses—for instance, by starting with social network data to select individuals in brokerage positions to consecutively gain more in-depth information from qualitative data (i.e., Spillane & Kim, 2012). Last, there was one conceptual paper in the studies selected for this review (Cooper et al., 2020).

#### Identifying brokers and brokerage

In all qualitative studies, brokers were identified in advance from a normative standpoint: A stakeholder's specific role (such as professional intermediary or teacher educator) was decisive for analyzing their brokering activity. However, whether these stakeholders acted as brokers or if brokering activity used other more informal paths was often neglected. In studies that applied social network analyses, three different measures were used to identify brokers or brokerage:

- Betweenness-centrality (*n* = 9), which indicates an actor’s centrality based on the shortest paths when information flows from one end to the other within a social network (Freeman, 1977)
- A more formal approach, proposed by Gould and Fernandez (1989), to analyze potential types of brokerage behavior (coordinator, itinerant, gatekeeper, representative, or liaison manager) related to group affiliation and direction of brokering activities (*n* = 4)
- A more structural approach using exponential random graph modeling (Lusher et al., 2013), where the presence or absence of ties (in this case, brokerage ties bridging structural holes) was statistically modeled and compared to simulated random network structures (*n* = 1)

### Stakeholders involved in brokering activity (RQ3)

**Fig. 2:**
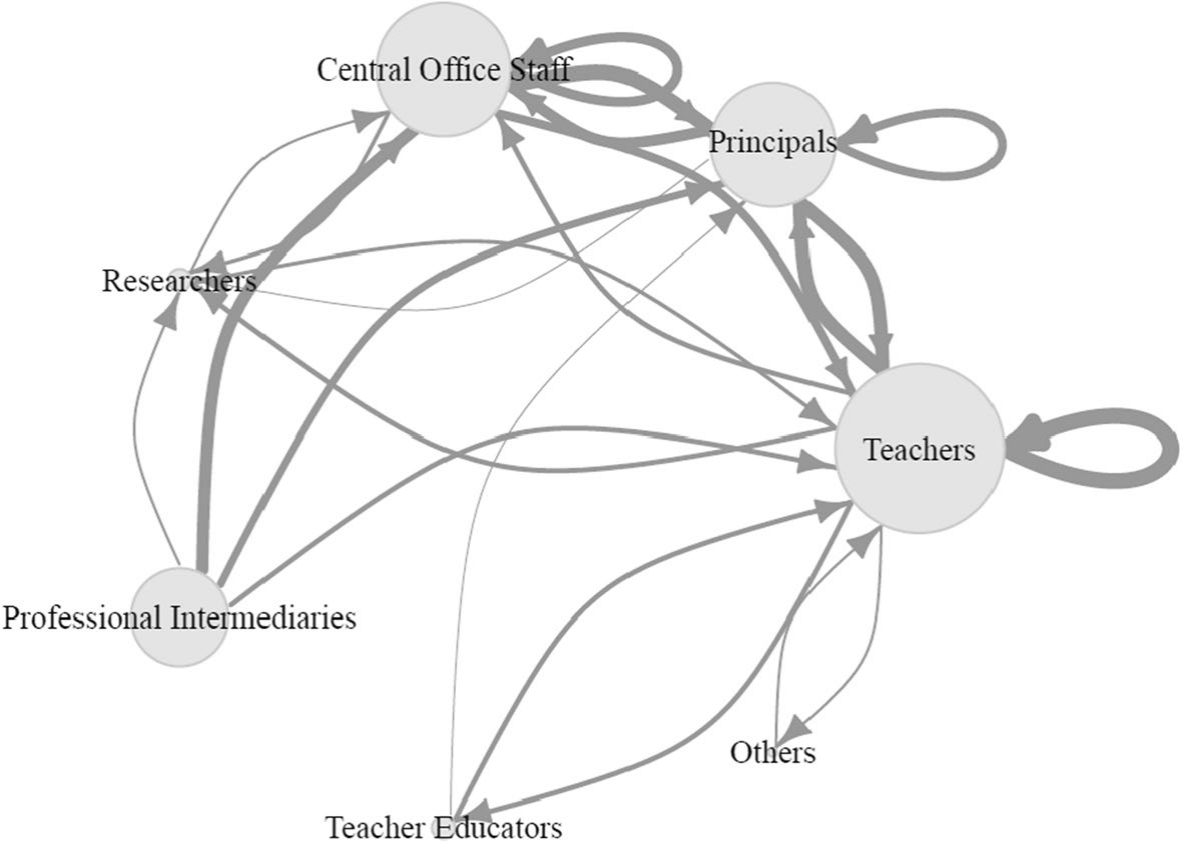
Content network of the stakeholders involved in brokering activities in the selected studies. Ties indicate in what direction brokering activities were analyzed in the selected studies (*N* = *42*). Nodes represent the different stakeholders involved in brokering activities. Weight of the ties represents the number of studies analyzing a specific gap being bridged *(Min* = *1; Max* = *13)*. Size of the nodes depends on the out-degree of centrality (based on out-going ties), indicating how often stakeholders were analyzed as brokers

Fifteen studies examined brokerage between different levels (cross-level: such as between the system and the school level), and 27 studies analyzed brokerage within a specific level (within-level: such as within the school level), illustrating the complex architecture of the loosely coupled and multilevel nature of educational systems (Creemers & Kyriakides, 2007; Weick, 1976) (see Table 4). Further, our analysis revealed that a diverse spectrum of stakeholders is involved in brokering activities. The actors analyzed the most frequently were practitioners of the school staff (such as teachers and school principals) and central office staff. Other relevant actors in brokering activities are external stakeholders such as professional intermediaries, researchers, and teacher educators. The content network graph in Fig. 2 shows that teachers were the most often analyzed targets of brokering activity. If teachers were analyzed as brokers, their targets would be mostly other teachers or the principal. Principals themselves are well-positioned between the teachers and the central office staff. The work of professional intermediaries was most prevalent in research on the interaction with the central office staff. As researchers interacted with almost all the other stakeholders, it is noteworthy that the studies analyzed them much more often as targets of brokering than as brokers themselves. The most important stakeholders were:

- actors on the class and school level (teachers and principals)
- actors on the system level (central office staff)
- external experts (professional intermediaries, researchers, and teacher educators)

However, there were two other actors that were analyzed as stakeholders, the staff of an aquarium (Kisiel, 2010) and art brokers as outside experts supporting the implementation of a more collaborative practice among teachers and artists (Fahy & Kenny, 2022).

### Empirical findings on brokerage in educational change (RQ4)

**Fig. 3:**
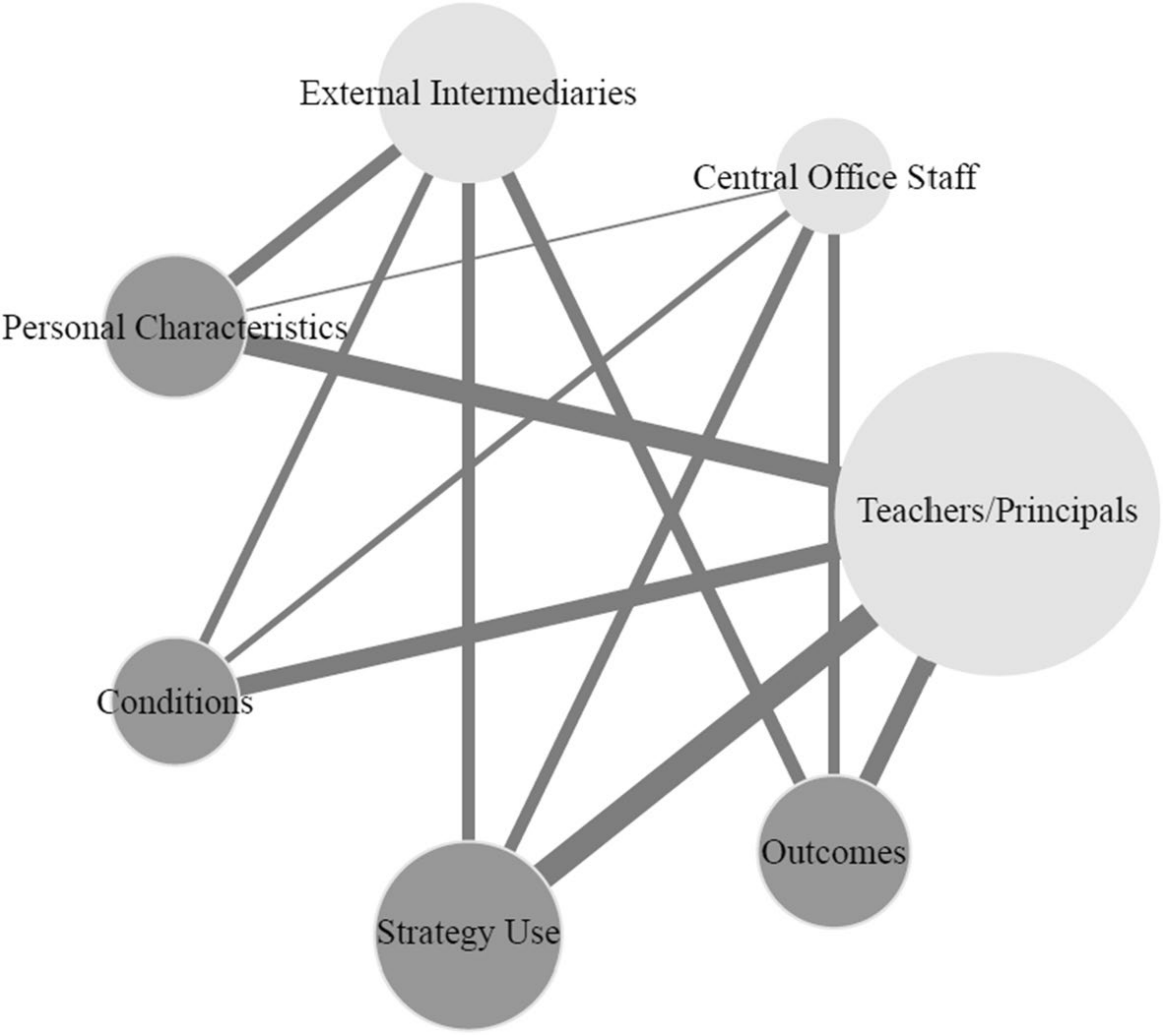
Content network: Aspects of empirical evidence in the selected studies related to the different stakeholders. Undirected ties indicate associations between four aspects of empirical evidence identified in the selected studies (*N* = *42*) related to the different stakeholders. The light gray nodes represent the different stakeholders; dark gray nodes indicate the different aspects of empirical evidence. Weight of the ties represent the number of studies with certain aspects related to a stakeholder *(Min* = *2; Max* = *20)*. Size of the nodes depends on the degree of centrality and indicates the frequency that the studies analyzed an aspect of empirical evidence and a specific stakeholder involved in educational change

Analysis of the selected studies in terms of empirical findings revealed a pattern of four relevant subcategories or aspects: (1) *personal characteristics* of the brokers, (2) *necessary conditions* that enable brokering activity, (3) successful *brokering strategies*, and (4) *outcomes* related to these activities on an individual and collective level. The content network graph (see Fig. 3) shows what evidence base was established for a specific stakeholder regarding these four aspects. All in all, most of the studies reported evidence on questions about what brokering strategies are used. Fewer studies examined necessary conditions to foster brokering activity. The following sections are organized by groups of stakeholders—teachers and principals, central office staff, and external intermediaries. Within each group, empirical evidence is outlined according to the four different aspects.

#### (A) Teachers and principals

Nearly half of the selected studies reported findings on the *personal characteristics* of brokers (*n* = 17). This evidence could be more consistent: For example, some studies argued that the presence or absence of attributes such as being experienced, trustworthy, open, honest, tolerant, sensitive, and communicative has a profound impact on whether one can act as a broker (Hopkins et al., 2013; Jusinski, 2021; van den Boom-Muilenberg et al., 2022), others pointed out that brokers do not have a specific profile in terms of attributes or working experience (Farley-Ripple & Grajeda, 2020). However, most studies agreed that different roles, mainly being in a leading position, impact the likelihood of finding oneself in a brokerage position. For example, some studies indicated that principals often tend to be in a sandwich position between teachers and central office staff and are well-positioned to act as brokers (Daly et al., 2014a; Park & Datnow, 2009). Moreover, principals more often than teachers saw themselves in brokerage positions (Park & Datnow, 2009).

Some selected studies reported on *necessary conditions* to foster brokerage (*n* = 14). Regarding these conditions, the studies often analyzed the three aspects of legitimacy, credibility, and trust. Some studies examined whether formal leading positions (such as principal) or informal leading positions are better for building bridges within their school teams. Whereas some studies found that informal leaders lack legitimacy to be brokers (Hopkins et al., 2013; Nordholm, 2016), others indicated that by formalizing brokers, credibility among teacher colleagues declines, and the effectiveness of brokering activity might be diminished (Jusinski, 2021). Social network analyses (Rechsteiner et al., 2022b; Spillane & Kim, 2012) revealed that formal organizational structures embracing a more shared leadership approach enhanced informal brokering activities by bringing highly credible staff members into a legitimate position to act as brokers. Bringing these two aspects of legitimacy and credibility together is always a matter of trusting each other—whether among colleagues or between principals and staff. Therefore, several studies indicated that a climate of trust is essential (Brown, 2020; Jusinski, 2021; Park & Datnow, 2009; Spillane & Kim, 2012; van den Boom-Muilenberg et al., 2022).

As outlined above, *brokering strategy use* can be conceptualized as a multidimensional process consisting of three dimensions: knowledge management, linking agency, and capacity building (Ward et al., 2009). In half of the studies (*n* = 20), we found evidence for these dimensions: First, different activities of knowledge management were analyzed, such as tailoring knowledge in a way that matches colleagues’ ability to process new information (Jusinski, 2021), crafting coherence about visions and goals of educational change across different groups of actors within and outside of school (Hopkins et al., 2013; LeChasseur et al., 2017), and introducing new boundary objects to negotiate meaning (Nordholm, 2016; Slavit & Roth McDuffie, 2013). Second, linking agency was identified, as brokers connect otherwise disconnected actors to facilitate staff interactions (Farley-Ripple & Grajeda, 2020; Hopkins et al., 2013; Park & Datnow, 2009). Third, capacity-building activities were revealed, as brokers empower other actors by scaffolding research and data use to further develop educational practice (Brown, 2020; Park & Datnow, 2009; van den Boom-Muilenberg et al., 2022; van Gasse et al., 2019; Wilkie, 2019).

By applying these strategies, principals in brokerage positions, in particular, can act as gatekeepers, buffering demands from central office staff (for instance, when implementing new policies) by being selective or even resistant to new reforms or innovations (LeChasseur et al., 2017; Zuckerman et al., 2018). Moreover, when participating in school networks, principals in brokerage positions can act simultaneously as representatives of their school by giving access to valuable information about their educational practice to a broader community and as gatekeepers by introducing new promising ideas from outside (Carmichael et al., 2006). Furthermore, successful brokering is about a principal’s ability to build trust and thoughtfully allocate resources (such as time and support) (Brown, 2020; Park & Datnow, 2009).

Teachers as brokers were described as floaters between different groups within a school (Spillane & Kim, 2012) and as go-to-experts (Hopkins et al., 2013). These descriptions indicated two possible directions: On the one hand, by floating from one group to another, they take on an active role by absorbing and sharing new ideas, materials, or perspectives. In this way, people on the periphery of different communities—are not necessarily always visible to others (Jusinski, 2021) and follow informal rather than formal organizational structures and roles (Rechsteiner et al., 2022a, b; Spillane & Kim, 2012; van den Boom-Muilenberg et al., 2022)—can act as brokers. On the other hand, a go-to expert is someone people reach out to for advice. Unlike a floater, an expert is more visible to other actors and often has more formal legitimacy (Hopkins et al., 2013; Nordholm, 2016).

More than every third selected study reported on different brokering *outcomes* (*n* = 14). Regarding individual outcomes, brokers’ networks tend to be larger and more diverse than their colleagues’ networks, which gives them better access to non-redundant information (Hopkins et al., 2013; van den Boom-Muilenberg et al., 2022). On an individual level, being a broker positively impacts motivation, confidence, and experience of autonomy (Hopkins et al., 2013; Slavit & Roth McDuffie, 2013). Additionally, brokers can locate and direct resources more actively to frame and direct their professional development (Slavit & Roth McDuffie, 2013). On the dark side, brokers risk overload and burnout more often, as they are vulnerable to exploitation on both sides of the gaps they are bridging (Jusinski, 2021).

Just a few studies (*n* = 5) reported on outcomes on a collective, organizational level. Evidence shows that the quality of brokering activities varies significantly between schools and their staff (Hubers et al., 2018) without specific reasons. There is also the risk that unstructured and informal brokerage might end up in a communication chain equivalent to the game of telephone, in which information flow is out of control and content may change arbitrarily (Dalyet al. 2014a). Moreover, in terms of acting as a multiplicator for professional development, Nordholm (2016) found that only the broker’s personal capacity is often enhanced, leaving organizational capacity unaffected. One last problematic aspect mentioned is that often the actors most in need of access to valuable information and support (such as principals in weak schools) are the least likely to be involved in brokering activity (Daly et al., 2014a, b). Eventually, following the argumentation of the strength of weak ties (Granovetter, 1973), schools and their staff might profit from brokerage constellations by having access to non-redundant information, which makes them more innovative and gives them an information advantage to anticipate better upcoming changes or challenges (Farley-Ripple & Grajeda, 2020; van den Boom-Muilenberg et al., 2022).

#### (B) Central office staff

There was little research reporting evidence on the *personal characteristics* of central office staff (*n* = 2). However, central office staff using their potential to bridge gaps were described as system travelers (Swinnerton, 2007). By moving up and down flexibly in the complex and multilevel educational system, central office staff acting as brokers will most likely understand different stakeholders’ contexts (Davidson & Penuel, 2020).

Five studies pointed out that trust is a *necessary condition* when central office staff reaches out to other stakeholders. New staff members have reported feeling uneasy about acting as brokers, as knowing different stakeholders’ contexts and experiences seems essential for establishing mutual trust among other stakeholders (Corbin et al., 2003; Hopkins et al., 2018).

Some selected studies (*n* = 9) reported evidence of central office staff involved in the *use of brokering strategies*: knowledge management, linking agency, and capacity building. For instance, knowledge management was identified as connecting and translating work between schools and the central office (Swinnerton, 2007) or identifying effective programs and services to support schools’ improvement (Daly et al., 2014a; Supovitz, 2008). Linking agency was identified as the central office staff’s potential to connect stakeholders both internal and external to the school system (Durand et al., 2015) to create a shared problem space (Davidson & Penuel, 2020), where coherence between various actors in educational systems can be crafted (Hopkins et al., 2019; Swinnerton, 2007).

Finally, capacity building was also addressed in supporting schools to improve educational practice (Hashim, 2020; Supovitz, 2008).

Almost every fourth study reported *outcomes* of brokering activities involving central office staff members (*n* = 7). However, there was almost no evidence of any outcomes on an individual level. A few studies indicated that central office staff often leave their potential to be brokers unexploited (Daly et al., 2014a, b; Hopkins et al., 2018). By delegating brokering activities to intermediary organizations, district leaders risk losing credibility as educational experts (Supovitz, 2008). Thus, Daly et al., (2014a, b) found a need for coherence between what district leaders are mandated to do to support schools and what they do. On a collective level, proactive and adaptive district leaders anticipate reforms early on and have the potential to buffer other stakeholders from external demands (Durand et al., 2015) and disruptive innovations (Zuckerman et al., 2018). Some studies (*n* = 3) indicated that central office staff, in some cases, did not take advantage of their potential role as gatekeepers (Durand et al., 2015; Hashim, 2020), revealing that they were especially reluctant to buffer external demands on practitioners. This situation can be even exacerbated by district leaders acting as liaison brokers, connecting external stakeholders and their demands to practitioners in an unfiltered way (Neal et al., 2019).

#### (C) External experts

More than every fourth study reported on external intermediaries’ characteristics (*n* = 10). It is not trivial to list the *personal characteristics* of external intermediaries, because compared to the stakeholders mentioned above, they tend to be a complex set of actors: i.e., professional intermediaries, researchers, or teacher educators. Even among the professional intermediaries, there are multiple subgroups (such as individuals or organizations, non-profit or profit, non-governmental or governmental institutions) pursuing various functions (such as raising awareness among practitioners for new trends or engaging them in capacity-building processes) that need to be distinguished (Cooper et al., 2020). It was pointed out that it is not entirely clear who they are, on what behalf they act, what they do, and what makes them effective (Cooper, 2012). However, there was a growing awareness that they have increasingly influential roles in disseminating research evidence in education (Cooper, 2012). Furthermore, some studies addressed potential threats of influential think tanks, clearing houses, or influencers following a (sometimes hidden) political or personal agenda in an attempt to influence practitioners in their decision-making (Jabbar et al., 2014; Malin & Paralkar, 2017; Morel & Coburn, 2019). Moreover, teacher educators, as a second kind of external experts, were described as brokers that shift their role from co-researcher, co-coach, or co-mentor, to co-learner, depending on the situation and aim of brokerage (Willegems et al., 2016). As teacher educators are actors that often have a background in practice and research, they were reported to be exceptionally well-placed to act as intermediaries (Ng-A-Fook et al., 2015; Sharples & Sheard, 2015).

A few studies focused on *necessary conditions* for external intermediaries (*n* = 7). It was revealed that one essential condition for external experts in brokerage positions is to consider the bidirectional nature of bridging gaps. For instance, it is not only the researchers or teacher educators who influence practitioners but also the other way around (Ng-A-Fook et al., 2015; Wilkie, 2019). Therefore, long-lasting trust-based relationships must be established (Kubiak, 2009). Brokerage with external stakeholders was, therefore, often described as an iterative process in which ongoing dialogue is necessary to understand the different contexts, organizational structures, and practitioners’ most pressing challenges to avoid possible pitfalls and steer clear of unnecessarily destabilizing well-working routines (Sharples & Sheard, 2015). In line with that, some researchers argued that the ability of external experts to create a shared problem space where meaning can be constantly negotiated is decisive for the success of their impact on educational change (Sharples & Sheard, 2015; Willegems et al., 2016). However, if they are involved in brokering activities, external experts often take over the active part, not seldom neglecting the reciprocal nature of brokerage (Neal et al., 2019).

*Brokering strategies* were reported in nine studies, which described successful brokering as offering a service that matches the interests and skills of the recipients. As practitioners often need more time and skills to process detailed and highly technical research, from the standpoint of educators and central office staff, brokering might result in packaging research evidence in easily digestible portions (Jabbar et al., 2014). Thus, in a best-case scenario, external experts can match research evidence to the demands and skills of their recipients, therefore bridging the research to practice gap, which should result in better educational practice and, eventually, improved student outcomes. Moreover, external experts have the potential to act as network facilitators (Kubiak, 2009). In this role, they connect otherwise disconnected actors in the broader network. Finding anchors in different networks to whom issues of immediate professional concerns can be addressed (Hashim, 2020; Kubiak, 2009) is, therefore, one crucial activity of external experts. This way, practitioners are presented with unfamiliar perspectives on their daily practice. As brokering activities are often associated with a feeling of threat, external experts—with their expertise and in their position—have the potential to scaffold how to deal with uncertainty (Wilkie, 2019). To this end, they need to know about the complexity of educational landscapes and their actors, the demand and supply of research evidence, and potential challenges when connecting communities (Kubiak, 2009; Wilkie, 2019).

Several selected studies focused on brokerage *outcomes* when external experts are involved (*n* = 10). However, these studies provided scant evidence of successful brokerage. One reason for this is that when external stakeholders are introduced to practitioners, lines of demarcation between different communities start to shift, which is often associated with critical dynamics (Chang, 2020; Willegems et al., 2016), and questions arise about the legitimacy and credibility of new actors involved in brokering activity. Positive outcomes were only reported regarding teacher educators using their potential to be brokers (Cooper, 2019; Wong, 2012). Being highly credible as educational experts and having enough legitimacy to act as change agents, teacher educators can change the rationale of teaching (Wong, 2012) and support capacity building of teachers’ scientific reasoning (Cooper, 2019). Especially in the role of co-mentors, teacher educators seem to be the most effective when changing educational practice (Willegems et al., 2016; Wong, 2012). Moreover, some researchers pointed out that delegating brokering activities—such as implementing a new policy or curriculum reform, or introducing an innovative teaching idea—to professional intermediaries does not come without a price. Educators and central office staff risk losing credibility and their authority as experts regarding issues related to teaching and education (Chang, 2020; Jabbar et al., 2014; Supovitz, 2008).

## Discussion

This study aimed to examine how the brokerage concept has been applied in research on educational change. The results of the analyzed 42 articles indicate that brokerage is a theoretical lens with a high potential for better understanding educational change. Here we discuss the key contributions of this systematic review and then formulate a future research agenda based on the uncovered gaps. Finally, we outline the limitations of this study.

### Key contributions of this study

As a *first contribution*, this review shows that brokerage is, by its origin, an interdisciplinary concept. The multitude of theoretical frameworks is a strength of the concept rather than a limitation. Thus, efforts to create a single framework for brokerage in the educational context might not be purposeful, because the educational landscape is complex and multilayered. Therefore, it is not a question of finding a brokerage theory that fits all but of applying a theoretical framework in correspondence with the aim of a study. Up to now, there is a preponderance of studies based on knowledge mobilization frameworks, followed by a large margin by Wenger's social learning theory on “communities of practice.” These two frameworks are primarily concerned with challenges associated with transferring knowledge across gaps between groups of actors.

Moreover, research on educational leadership has also taken up the brokerage concept. However, only a few studies used social capital and distributed leadership frameworks. They are both primarily concerned with analyzing power distribution and comparing formal to informal organizational structures and roles when collectively developing educational practice. The selected studies vary in the theoretical assumptions concerning brokerage and also in their use of terminology. Although we decisively oppose a single theoretical brokerage framework, consistent use of the terminology is necessary. Following the argumentation of Farley-Ripple (2019), we suggest applying brokerage as the key terminology in future research because it entails both brokers and brokering, emphasizing the relation between the two. Moreover, according to Malin and Brown (2020) using the term brokerage allows researchers to focus on the complex social interaction rather than an individualized phenomenon. Hence, using brokerage as key terminology may prevent future research from placing undue emphasis on individual brokers and their unilateral efforts, and thereby neglecting the multilateral and dynamic nature of the concept. In this regard, the overview in Table 3 provides a solid starting point when applying the brokerage concept in research about educational change.

As a *second contribution*, this study demonstrates teachers’ crucial role in further developing educational practice. In the studies selected, they were among the most analyzed. This stresses that some teachers, beyond the formal organizational structure, play an essential role in knowledge management, linking agency, and capacity-building processes at their schools. Therefore, emphasizing teachers as agents of change with the most leverage to improve educational practice at the core and addressing their potential to be active stakeholders in brokerage processes is most promising. In this respect, progress has been made in the last two years, with recent research explicitly addressing teachers as brokers (i.e., Rechsteiner et al., 2022a; Jusinski, 2021; van den Boom-Muilenburg et al., 2022).

Moreover, Rechsteiner et al., (2022b) examined the associations of different facets of brokerage in the form of teachers’ brokerage behavior, their formal entitlement to be a broker, and their structural position as a broker in the school’s network. As these facets only corresponded marginally, researchers need to carefully consider how to define and operationalize brokerage in a study. Another study examined teachers influencing policy generation from the bottom by applying a micro-political perspective (Giudici, 2021). These often-informal ways and hidden mechanisms of teachers actively generating new policies might be further illuminated by combining the two perspectives of micro-politics in education and brokerage.

Our *third contribution* is related to another stakeholder: teacher educators. They are reported to be potentially highly effective bridge-builders in further developing educational practice (for instance, bridging the research–practice gap) because of their background in research and practice. Future studies might further analyze their how they use their advantage in terms of credibility as a knowledgeable resource on educational issues and as having formal legitimacy to introduce new ideas or materials.

This study's *fourth and final contribution* is that no matter what stakeholders are involved and what gap is meant to be bridged, finding a balance between credibility and formal legitimacy is pivotal. Several of the studies indicated that the closer actors are to the classroom, the less formal legitimacy they have to act as brokers and the more likely they are to be targets of brokers. Hence, teachers’ lack of formal legitimacy is problematic, because the review shows they were among the most critical professional actors when brokering educational change. Formal legitimacy increases with actors further away from the classroom, such as central office staff, but simultaneously, the credibility and trust necessary for successfully mobilizing knowledge and building capacity decrease. Therefore, the potential for central office staff to act as brokers by mobilizing knowledge and working as linking agents or capacity builders is often left unexploited. In addition, external intermediaries play an increasingly dominant role in building bridges between practitioners and other stakeholders. As a result, central office staff risk losing their credibility as educational experts by relying on external intermediaries. In line with this, a recent case study by Malin (2020) found that policymakers risk being sidetracked by professional intermediaries. In this way, new actors may substantially influence educational change.

### Future research agenda

Based on this systematic review, we have identified six gaps in the current literature on brokerage and educational change:

1. Quantitative or mixed-method study designs with larger samples and more longitudinal research are rare. Instead, most studies applied qualitative study designs in small sample-size case studies, which do not allow generalizing the empirical evidence to a broader population.
2. More emphasis should be placed on the content and quality of advice and information exchanged by brokers (Hopkins et al., 2018; Morel & Coburn, 2019). For instance, Daly and Finnigan (2011) indicated that the quality of social relations is closely linked to changing educational practice more or less successfully. Therefore, future research might take a closer look at the content and the quality of the knowledge transferred and the brokering activities applied.
3. There is a need to differentiate the measures used to locate brokers in social networks. Betweenness-centrality has been the most frequently used measure up to now. This is not surprising, as betweenness-centrality is the most straightforward way to identify individuals in a brokerage position (Freeman, 1977). However, this approach has been criticized, as an actor’s influence beyond direct ties is rather theoretical and impractical. Gould and Fernandez (1989), for instance, argued that solely being multiple times on long paths from one end of the network to the other does not necessarily indicate “a very important role in purposive social interaction” (p. 95). Instead, keeping track of and using the complex social interaction patterns several steps beyond the people to whom an individual is directly connected seems unreasonable and impractical. Gould and Fernandez therefore suggested focusing exclusively on the direct ties connecting otherwise disconnected actors.
4. Brokerage is often analyzed as a rather static, unilateral, and individualized phenomenon. We suggest a shift to brokerage as “a dynamic and complex set of actors, activities, and motivations within which knowledge is exchanged, transformed, and otherwise communicated” (Farley-Ripple, 2019). Rather than viewing social network structure as determining whether an actor can act as a broker, network structures affect how a broker acts but do not define the way someone can build bridges between disconnected individuals (Rechsteiner et al., 2022b).
5. Few studies focus on ways to foster optimal conditions and potential pitfalls that constrain brokering activities. In the studies reviewed here, several researchers raised the question as to how brokers can be supported to be successful change agents (Rechsteiner et al., 2022a; Farley-Ripple & Grajeda, 2020; Willegems et al., 2016), mentioning no more current idea than the buffer zone for all stakeholders involved in brokering activity proposed by Kubiak (2009) more than a decade ago. Thus, more research is needed that examines the organizational features with the most significant effects on successful knowledge mobilization or capacity building among practitioners.
6. On both an individual and a collective level, little attention has been paid to the dark side of brokerage (Hopkins et al., 2018). On an individual level, research outside education suggests that brokers often suffer from intense pressure, as sustaining bridges can be very demanding (Krackhardt, 1999). For instance, there can be tensions when simultaneously exercising multiple aspects of brokerage (Kislov et al., 2017), such as tensions between different aspects of brokering, tensions between different types and sources of knowledge, and tensions between the group of actors. So far, only one study in this review (Rechsteiner et al., 2022b) indicated that brokerage and negative affect (work-related stress) are not correlated. On a collective level, previous research indicated that brokers could be significantly (sometimes even too) powerful in controlling the flow of information in a network (i.e., Krackhardt, 1999): First, in their function as bottlenecks, brokers can hoard and distort information. Second, brokers are sometimes formally appointed to their positions. Making informal power structures visible and trying to change them is often challenging. Third, if a broker leaves an organization, a network might even collapse. Future research might elaborate on these aspects of the dark side of brokerage in educational change.

### Limitations

With the 42 articles reviewed here, there are, up to now, only a limited number of studies examining brokerage explicitly within the research domain of educational change. Therefore, any generalization of our findings must be considered cautiously. As we included only English-language articles, most studies were conducted within a Western cultural context (91%). However, it is reasonable to assume that there are cultural differences in terms of brokerage, primarily related to the issues of legitimacy and credibility of being in a brokerage position, but also in terms of power structures among the stakeholders involved.

Another limitation of this study is that it explicitly focused on brokerage, brokers, and brokering. We argue that this is instrumental in accurately describing the use of the constructs for the first time in the context of educational change. However, we acknowledge that other strands of research address similar issues and use related constructs, such as boundary-crossing or the work of multiplicators (i.e., Jesacher-Roessler, 2021; Rycroft-Smith, 2022). In this regard, Rycroft-Smith (2022) provides an excellent overview of different conceptualizations and metaphors when studying knowledge mobilization (such as knowledge brokering or boundary spanning). To extend her work, we would like to highlight some other well-established concepts with a distinct emphasis on educational change that could benefit from convergence with the brokerage concept:

For example, in their work on teacher research, Cochran-Smith and Lytle (2015) indicated that establishing communities for teacher research is essentially about restructuring teachers’ social interaction patterns in their school and community contexts. The brokerage concept may aid in studying teachers’ relationships in or beyond their schools. A further approach is the “dynamic approach to school improvement” in Kyriakides et al. (2019), where researchers act as brokers, although not yet labeled as such. Teachers as change agents or opinion leaders (Hemsley-Brown & Sharp, 2003) are other relevant concepts that might benefit from the brokerage concept. Future research might elaborate further on the overlap, discrepancies, and mutual potential of brokerage with other concepts.

## Conclusion

For quite some time, research outside the field of education has indicated the potential of the brokerage concept for studying organizational change processes. This study aimed to spark discussion on this concept in educational change. We believe that the interdisciplinary origins of brokerage outlined in this study are fertile soil for framing the concept theoretically and approaching it methodologically in a context-sensitive way—be it for teachers, principals, central office staff, or any other relevant stakeholders involved in changing educational practice. Finding a balance between these stakeholders’ credibility and formal legitimacy when educational practice is to be changed seems to be not only essential but also a highly challenging endeavor. Thus, more research on brokerage in educational change might contribute to our understanding of bridging gaps more successfully, making sustainable improvements at the core of educational practice more and more the rule than the exception.
